# P05.43. Meta-ethnography: the perspective of patients choosing alternative and complementary medicine regarding individualized medicine and integrative care

**DOI:** 10.1186/1472-6882-12-S1-P403

**Published:** 2012-06-12

**Authors:** B Franzel, P Heusser, R Lauche, M Schwiegershausen, B Berger

**Affiliations:** 1 University of Witten/Herdecke, Center for Integrative Med, Herdecke, Germany; 2 Univ Duisburg-Essen Klinik für Naturheilkunde und Integrative Medizin, Essen, Germany

## Purpose

Personalized medicine in the days of genetic research is seen as molecular biologic specification in individuals, not as individualized care oriented to patients needs in the sense of person-centered medicine. Yet the question can be raised whether this focus can ameliorate health care needs in view of the invested resources. Studies suggest that patients often miss authentically patient-centred care and individual physician-patient interaction and therefore decide to choose complementary and alternative medicine (CAM). By means of a meta-ethnography this project explored patients’ views about individualized medicine and described the patients’ perspectives of integrative and person-centred services.

## Methods

The procedure included first an electronic databases search for ‘qualitative research’ AND ‘CAM’ AND ‘patient expectations’ subject headings, supplemented with citation searches and hand searching. Second, studies were assessed using an inclusion/exclusion checklist and a quality score according to an adjusted checklist of Behrens and Langer. The third step was meta-ethnography according to Noblit and Hare's method to synthesize and interpret key concepts of individualized medicine using a line of argument synthesis.

## Results

A set of 67 electronic databases including CAM, nursing, nutrition, psychological, social, medical databases, the Cochrane Library and DIMDI were searched. Nine thousand five hundred seventy-eight citations were screened, 63 full text publications reviewed, 38 appraised and 30 articles were included. The third-order constructs emerging through the synthesis and interpretation conducted by the multidisciplinary research team were: "Personal development", "holism", "alliance", "room for connecting different models", "self activation", "dimensions of well-being".

## Conclusion

The perspective of patients choosing alternative and complementary medicine regarding individualized care clearly differs from the current idea of personalized genetic medicine. Individualized medicine has therefore concurrently to bear in mind the humanistic approach of holistic, transformative, integrative and self-activation needs of patients. The allocation of resources should consider patients needs to enhance a high-quality biomedical or scientific health care system.

